# Linezolid in combination with pentazocine causes serotonin syndrome: A case report

**DOI:** 10.1097/MD.0000000000043517

**Published:** 2025-07-18

**Authors:** Xie Xinpeng, Wei Xiaoting, Lin Sheng

**Affiliations:** a Department of Surgical Intensive Care Unit, Yantaishan Hospital Affiliated to Binzhou Medical University, Yantai, Shandong Province, China.

**Keywords:** linezolid, pentazocine, serotonin, serotonin syndrome

## Abstract

**Rationale:**

Serotonin syndrome (SS) is a potentially life-threatening condition characterized by autonomic and muscular hyperactivity resulting from the use of serotonergic drugs that bind to peripheral or central postsynaptic serotonin receptors. This syndrome can be easily confused with other clinical conditions, leading to delays in diagnosis and jeopardizing vital prognosis. Linezolid is utilized in the clinical management of gram-positive coccal infections, while pentazocine, an opioid analgesic, is frequently employed for pain relief in trauma patients. Currently, there are limited reports documenting the occurrence of SS associated with linezolid and pentazocine. In this report, we present the case of a 32-year-old man who developed SS following simultaneous administration of linezolid and pentazocine; his symptoms were effectively managed upon discontinuation of these medications.

**Patient concerns:**

A 32-year-old male patient was treated with pentazocine and linezolid for pain management related to trauma as well as for a gram-positive coccal infection. One hour after administration, he exhibited tremors in his right limb along with hand tremors, profuse sweating, and sinus tachycardia; his body temperature subsequently rose to 40°C. Laboratory tests including white blood cell count, procalcitonin levels, and C-reactive protein, showed no significant changes compared to previous results. A follow-up computed tomography scan of the head revealed no new ischemic lesions.

**Diagnoses:**

The patient’s physical examination, vital signs, and laboratory results were consistent with SS.

**Interventions:**

Linezolid and pentazocine were discontinued immediately. Midazolam (3–6 µg/kg/h) and dexmedetomidine (0.1–0.3 µg/kg/h) were administered, and oral diazepam (2.5 mg 3 times daily) was gradually continued.

**Outcomes:**

Following this treatment regimen, the patient’s profuse sweating improved significantly, and he became stable overall; his body temperature gradually returned to normal levels. Subsequently, the patient was transferred from the surgical intensive care unit to a general ward after 7 days of treatment.

**Lessons:**

Although SS is not very common in patients using pentazocine and linezolid separately, it is important because it is an emergency condition that can result in death if not treated appropriately. This clinical case highlights the importance of thoroughly understanding the clinical manifestations of SS to ensure early and appropriate treatment management.

## 1. Introduction

Serotonin syndrome (SS), resulting from an excessive accumulation of serotonin in the central nervous system, can occur due to various factors such as the initiation of medication, overdose, or drug interactions. This syndrome has 3 main clinical features: (a) neuromuscular hyperactivity, such as tremor, myoclonus, and hyperreflexia; (b) autonomic hyperactivity including diaphoresis, fever, and tachycardia; (c) altered mental status that could present with agitation and confusion.^[1]^ Most documented cases of SS are in patients taking multiple serotonergic drugs or who have had considerable exposure to a single serotonin-augmenting drug.^[2]^ Linezolid, a synthetic oxazolidinone antibiotic, is used to treat gram-positive bacterial infections, including methicillin-resistant *Staphylococcus aureus* (MRSA). Although linezolid can lead to SS, it is not very common, and cases caused by its concurrent use with other medications are even rarer. Pentazocine, as a weak opioid, may slightly inhibit monoamine oxidase (MAO) and reduce serotonin breakdown. When used in combination with other serotonergic drugs, these mechanisms can lead to excessive serotonin accumulation. Currently, there are no reported cases indicating that it can cause SS. With the increase in publications on cases of SS, the data obtained will contribute to providing the information necessary for a more accurate clinical approach for this group of patients.

## 2. Case presentation

**Figure 1.**
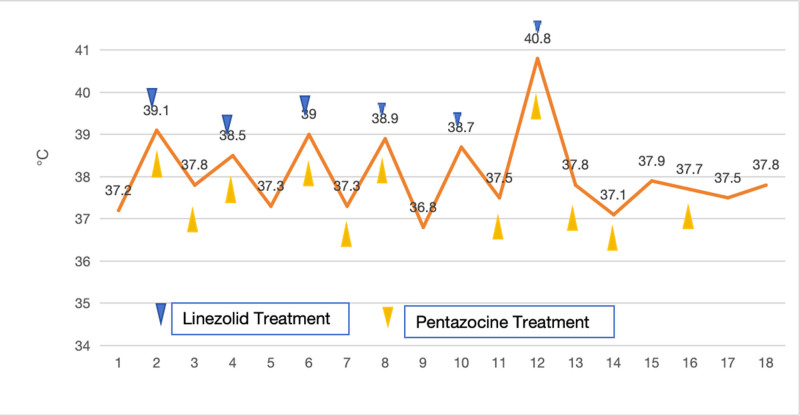
The trend in body temperature changes following the administration of pentazocine and linezolid. As shown in the figure above, the patient’s body temperature changes during the application of linezolid and pentazocine. The blue and yellow arrows indicate the body temperature after the application of linezolid and/or pentazocine, respectively. The numbers 1–4, 5–8, 9–12, 13–15, 16–18 represent the temperature changes from December 20 to 24, respectively.

**Figure 2.**
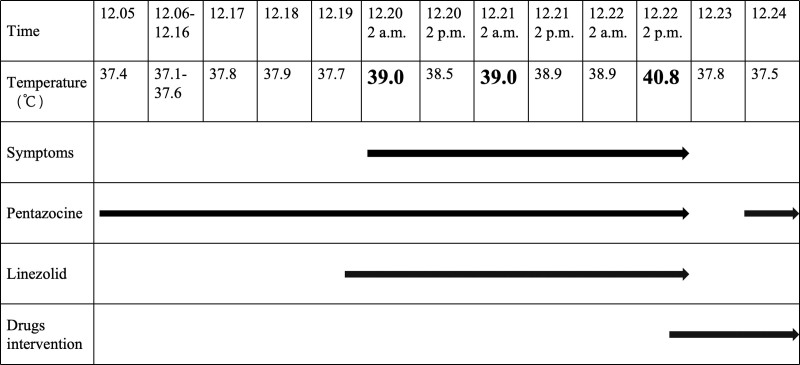
Record of body temperature variations and timing of drug intervention. As shown in the figure above, the body temperature section illustrates the trend of body temperature fluctuations from admission to discharge. Symptoms include profuse sweating and muscle tremors, and sinus tachycardia, among others. Drug interventions encompass the administration of dexmedetomidine (0.1–0.3 µg/kg/h), midazolam(3–6 µg/kg/h), and diazepam (2.5 mg 3 times daily). The black pointed tip denotes the duration of continuous treatment or observation.

A 32-year-old male was admitted to the surgical intensive care unit on December 5, 2024, with traumatic brain injury and an open right-leg wound. This patient had no previous history of mental illness or drug use that might cause SS. Initial management included mechanical ventilation, wound debridement, and pentazocine (30 mg intramuscularly, 3 times daily) for pain control. Blood cultures later revealed MRSA, prompting vancomycin therapy (1 g IV every 12 hours). On December 19, follow-up showed elevated creatinine levels compared with admission, along with increased potassium levels and a trend of decreased urine output. To prevent renal failure caused by vancomycin, the medication was discontinued, and linezolid 600 mg IV every 12 hours was initiated for infection treatment. There were no adverse reactions on that day. On December 20, after receiving pentazocine 30 mg, he underwent debridement and dressing change of the lower limb. About an hour after debridement and approximately 2 hours after linezolid infusion began, the patient experienced tremors in the right limb, hand tremors, profuse sweating, and sinus tachycardia. After administering esmolol, the heart rate was controlled; however, the patient’s physical symptoms did not improve, and his temperature increased to 39°C. Immediate measures were taken, including physical cooling with an ice blanket and drug-induced cooling with indomethacin suppositories, which led to a gradual decrease in temperature. On December 21, the same symptoms recurred during the linezolid administration. White blood cell count, procalcitonin, and C-reactive protein were rechecked, showing no significant changes compared to previous results. On December 22, after 2 additional doses of linezolid, the patient again exhibited the same symptoms, with his temperature further rising to 40.8°C (Table 1). Thyroid function tests were conducted immediately again on the same day, and the results showed no significant change compared with those at admission. A follow-up computed tomography scan of the head revealed no new ischemic lesions. A lumbar puncture was conducted, with routine analysis and cultures sent for testing, along with procalcitonin and other tests, to rule out intracranial infection. The patient received daily arterial blood gas analysis and monitoring, with no abnormal electrolyte levels detected. After reorganizing the patient’s medication orders and considering the pattern of fever and medication timing, we suspected that the overlap of linezolid and pentazocine administration contributed to the symptoms of SS (Figs. 1 and 2). Linezolid and pentazocine were discontinued immediately. As the blood culture results were negative, no further treatment for gram-positive cocci was initiated. Midazolam (3–6 µg/kg/h) and dexmedetomidine (0.1–0.3 µg/kg/h) were administered, and oral diazepam (2.5 mg 3 times daily) was gradually continued. One day after drug withdrawal, the patient no longer exhibited symptoms, such as tachyarrhythmia, profuse sweating, hand tremors, muscle spasms, or malignant hyperthermia. Owing to multiple debridements of the lower limb, pentazocine was continued for pain management, and no SS-related symptoms were observed after its administration. Ultimately, the patient was discharged from the hospital smoothly, and none of the above symptoms were observed in the subsequent follow-up investigation. The patient’s condition and the corresponding intervention measures are illustrated in Figure 2.

**Table 1:** Blood laboratory results within 5 days after application of linezolid.

	Day 1	Day 2	Day 3	Day 4	Day 5
White blood cells(3.5–9.5 × 10^9^/L)	9.86	9.24	10.76	7.60	7.09
C-reactive protein(0–10 mg/L)	104.31	99.15	97.75	115.34	109.56
Procalcitonin(0–0.04 ng/mL)	2.19	1.90	2.10	1.26	0.80
ALT/AST(9–50 U/L; 15–40 U/L)	66/67	-	177.5/174.9	-	-
Sodium potassium ion (137–147 mmol/L; 3.5–5.3 mmol/L)	136.7/4.42	135.7/4.24	133.5/4.74	136/4.35	-

## 3. Discussion

Linezolid is an oxazolidinone antibiotic and a reversible nonselective monoamine oxidase inhibitor (MAOI). It can inhibit bacterial growth and reproduction, particularly against resistant gram-positive cocci such as MRSA or vancomycin-resistant Enterococcus infections. Given its action as an MAOI, it can increase serotonin levels. Data from a randomized controlled trial involving over 5000 patients indicated that the incidence of SS with linezolid was 0.54% compared with 0.19% in the control group. Although there was no significant difference between the 2 groups, the results suggested a slightly higher incidence of SS in patients receiving linezolid.^[1,3]^

Serotonin is an important neurotransmitter involved in the regulation of physiological activities such as sleep, emotional behavior, appetite, body temperature, vomiting, and sexual activity. SS is a drug-related complication that occurs when 2 or more serotonergic drugs are concurrently used, leading to drug interactions that result in excessive serotonin release or receptor blockade. SS was diagnosed via Hunter Criteria,^[4]^ if one of the following events happened: (1) spontaneous clonus; (2) inducible clonus plus agitation or diaphoresis; (3) ocular clonus plus agitation or diaphoresis; (4) tremor and hyperreflexia; (5) hypertonia; and (6) temperature above 38°C plus ocular clonus or inducible clonus. SS was also diagnosed via Radomski criteria,^[5]^ if at least 4 major or 3 major plus 2 minor variables developed. Major criteria include (1) mental findings (consciousness impairment, elevated mood, and semicoma/coma); (2) neurological symptoms (myoclonus, tremor, shivering, rigidity, and hyperreflexia); (3) vegetative symptoms (fever and sweating). Minor criteria include (1) mental findings (restlessness and insomnia); (2) neurological symptoms (incoordination, dilated pupils, and akathisia); (3) vegetative symptoms (tachycardia, tachy/dyspnea, diarrhea, and hyper/hypotension). Symptoms typically arise within 6 to 8 hours after administration,^[6]^ and clinical diagnosis refers to the criteria proposed by Dunkley and Radomski: taking serotonergic drugs within the past 5 weeks, with the presence of any of the following symptoms: (1) tremors, hyperreflexia; (2) spontaneous clonus; (3) muscle rigidity, temperature >38°C, ocular clonus, or inducible clonus; (4) ocular clonus, agitation, or sweating; and (5) inducible clonus, irritability, rigidity, or sweating. Other causes of the symptoms must be ruled out.^[7]^

The main differential diagnoses of SS include malignant hyperthermia, alcohol or drug (benzodiazepine) withdrawal, other drug toxicity, thyroid diseases, electrolyte disorders, central nervous system infections, and other infectious diseases. The attending physician did not find any history of drug use or excessive alcohol consumption after reordering the medical order; the patient had no underlying thyroid disease; thyroid function review and ultrasound examination during hospitalization showed no abnormal findings; electrolyte level during hospitalization showed no abnormal findings; the nature of lumbar puncture fluid, related laboratory tests, bacterial culture, and other results did not support central nervous system infection. During hospitalization, the patient underwent repeated chest and abdominal computed tomography examinations and bronchoscopy treatment, and the open wound of the lower limb was effectively debrided, and the dressing was changed. The results of blood culture and sputum culture during high fever were negative, and the indices of white blood cells and procalcitonin did not increase significantly. The above findings were significantly different from the clinical manifestations of high fever; therefore, infectious lesions were not supported.

SS can be classified as mild, moderate, or severe according to clinical symptoms. In severe cases, severe hypertension, tachycardia, and hyperthermia (body temperature > 41°C) usually result in sudden shock, agitated delirium, myotonia, seizures, etc. The auxiliary examination showed elevated aminotransferase and creatinine levels, metabolic acidosis, diffuse intravascular coagulation, rhabdomyolysis, and renal failure.^[8]^ The patient had an open wound in the lower extremities caused by trauma. Owing to severe pain, pentazocine was routinely administered 3 times a day for pain relief, and no signs of SS appeared. Because blood culture indicated MRSA and abnormal renal function, linezolid anti-infection treatment was administered, and pentazocine, as one of the opioids combined with linezolid, significantly increased the risk of SS.^[9]^ Within about 2 hours after intramuscular injection of pentazocine in advance and administration of linzolid, the patient developed a high fever with a body temperature of 40.8°C (Fig. 1), accompanied by binocular staring, intermittent tremor of the right limb, muscle rigidity, sudden rise of blood pressure, rapid heart rate, and heavy sweating. Trauma resulted in a large cerebral infarction on the right side and immobility of the left limb. Therefore, the left limb tremor did not appear, and the patient’s medication history, symptoms, signs, and time sequence of the 3 occurrences were in line with the clinical diagnosis criteria of SS.

Most reported SS cases involve selective serotonin reuptake inhibitors or MAOIs and at least one other serotonergic drug.^[9]^ This mechanism is thought to be related to the overactivation of synapses, resulting from elevated levels of serotonin in the central nervous system and peripheral tissues. Linezolid is a weak MAOIs that prevents serotonin breakdown. Pentazocine, an opioid, can affect the concentration of serotonin in blood, which may be explained by opioid-mediated serotonin release. Opioids are widely used as analgesics in the clinical practice. Rachel reported a case of SS caused by the interaction between tramadol and pethidine.^[10]^ Tao et al^[11]^ found for the first time that systemic administration of fentanyl in mice receiving opioids resulted in a dose-dependent increase in serotonin. In this report, the patient required pain relief due to multiple injuries, and no adverse reactions occurred when pentazocine alone was used for pain relief. As the patient suffered from bacteremia simultaneously, linezolid antimicrobial treatment was required, and SS-related symptoms occurred when linezolid was administered. However, SS did not occur when linezolid was administered alone. After discontinuing the above drugs, the body temperature, heart rate, and blood pressure decreased gradually. The disappearance of limb symptoms, such as muscle tremors and rigidity, is considered to be related to the application of linezolid. At present, the specific molecular mechanism of SS induced by these drugs is not clear, and further research is needed to support this.

To the best of our knowledge, this is the first reported case of SS caused by linezolid combined with pentazocine. It is important for physicians to accurately identify SS based on medical history and patient clinical presentation when administering linezolid and pentazocine. Owing to the complex and varied signs and symptoms of this syndrome and the many similar differential diagnoses, this disease is easily overlooked. In addition, if SS is not recognized, the continued use of these drugs leads to worsening of the condition, which can have catastrophic consequences. Medications for SS include benzodiazepines and dexmedetomidine.^[12]^ The efficacy of cyproheptadine in the treatment of SS has been documented.^[13]^ Atypical antipsychotics with serotonin antagonist properties (such as olanzapine) have been attempted with some success.^[14]^

## 4. Conclusion

Linezolid, an antibacterial agent frequently used in the clinical management of gram-positive cocci infections, and pentazocine, an opioid analgesic, are effective in alleviating pain in patients suffering from major trauma. However, clinicians who may not be well-versed in the pharmacological properties and potential adverse effects of MAOI risk inadvertently induce SS when administering these opioid analgesics concurrently. This paper highlights a case of SS resulting from the combination of linezolid and pentazocine, which serves as a crucial reminder for healthcare professionals to remain vigilant regarding the possible interactions between these 2 drug classes. Timely cessation of implicated medications and early initiation of benzodiazepines or dexmedetomidine may be effective in managing this condition.

## Author contributions

**Conceptualization:** Lin Sheng.

**Data curation:** Lin Sheng.

## References

[1] Chiew AL Isbister GK. Management of serotonin syndrome (toxicity). Brit J Clin Pharmaco. 2023;91:654–61. 10.1111/bcp.16152 PMC11862804 38926083

[2] Ables AZ Nagubilli R. Prevention, recognition, andmanagement of serotonin syndrome. Am fam physician. 2010;81:1139–42. 20433130

[3] Butterfield JM Lawrence KR Reisman A. Comparison of serotonin toxicity with concomitant use of linezolid or comparators and serotonergic agents: an analysis of Phase III and IV randomized clinical trial data. J Antimicrob Chemoth. 2011;67:494–502. 10.1093/jac/dkr467 22139199

[4] Dunkley EJ Isbister GK Sibbritt D Dawson AH Whyte IM. Thehunter serotonin toxicity criteria: Simple and accurate diagnostic decision rules for serotonin toxicity. Qjm. 2003;96:635–42. 12925718 10.1093/qjmed/hcg109

[5] Radomski JW Dursun SM Reveley MA Kutcher SP. An exploratory approach to the serotonin syndrome. Med hypotheses. 2000;55:218–24. 10985912 10.1054/mehy.2000.1047

[6] El Mir K El Bouchalli W El Jabiry SE. Serotonin syndrome secondary to the association between paroxetine and amitriptyline: a case report. Pan Afr Med J. 2024;48:48–52. 39525544 10.11604/pamj.2024.48.109.44113 PMC11544001

[7] Badar A. Serotonin syndrome: an often-neglected medical emergency. J Family Community Med. 2023;31:1–8. 10.4103/jfcm.jfcm_236_23 PMC10883429 38406216

[8] Spadaro A Scott KR Koyfman A Long B. High-risk and low-prevalence diseases: serotonin syndrome. Am J Emerg Med. 2022;61:90–7. 36057215 10.1016/j.ajem.2022.08.030

[9] Essakow J Jin L Marupudi N Wattier R McQuillen P Franzon D. Serotonin syndrome associated with linezolid and opioid use in an infant. J Pediatr Pharmacol Ther. 2022;27:564–8. 36988981 10.5863/1551-6776-27.6.564 PMC9400179

[10] Gray R Iii A M Berry F. Serotonin syndrome after PACU administration of tramadol and meperidine. Turk J Anesthesiol Reanim. 2022;50:309–11. 10.5152/TJAR.2022.21355 PMC9524450 35979980

[11] Tao R Ma Z Auerbach SB. Alteration in the regulation of serotonin release in the rat dorsal raphe nucleus after prolonged exposure to morphine. J Pharmacol Exp Ther. 1998;286:481–8. 9655893

[12] Baumgartner K Doering AM Mullins ME. Dexmedetomidine in the treatment of toxicological conditions: a systematic review and review of the toxicology investigators consortium database. Clin Toxicol. 2022;60:1356–75. 10.1080/15563650.2022.2138761 36346349

[13] Murray BP Carpenter JE Sayers J. Two patients with serotonin syndrome after bupropion overdose were treated with cyproheptadine. J Emerg Med. 2020;60:67–71. 33308914 10.1016/j.jemermed.2020.10.039

[14] Savvidou A Jennions E Wikström S. Drug-induced hyperthermia with rhabdomyolysis in CLN3 disease. Eur J Paediatr Neuro. 2022;39:74–8. 10.1016/j.ejpn.2022.06.007 35716526

